# Scoping review of the World Health Organization’s underlying equity discourses: apparent ambiguities, inadequacy, and contradictions

**DOI:** 10.1186/s12939-021-01400-x

**Published:** 2021-03-03

**Authors:** Michelle M. Amri, Geneviève Jessiman-Perreault, Arjumand Siddiqi, Patricia O’Campo, Theresa Enright, Erica Di Ruggiero

**Affiliations:** 1 Dalla Lana School of Public Health, University of Toronto, 155 College Street, Toronto, Ontario M5T 1P8 Canada; 2 Takemi Program in International Health, Harvard T.H. Chan School of Public Health, 665 Huntington Avenue, Bldg. 1, Boston, MA 02115-6021 USA; 3 School of Public Health and Social Policy, Human and Social Development Building, University of Victoria, 3800 Finnerty Road, Victoria, British Columbia V8P 5C2 Canada; 4 Gillings School of Global Public Health, University of North Carolina - Chapel Hill, Chapel Hill, USA; 5 Li Ka Shing Knowledge Institute, St. Michael’s Hospital, 209 Victoria Street, Toronto, Ontario M5B 1T8 Canada; 6 Department of Political Science, University of Toronto, 100 St George Street, Toronto, Ontario M5S 3G3 Canada

**Keywords:** Equity, Equality, World Health Organization, Discourses, Theory of justice, Capabilities approach

## Abstract

**Background and objective:**

Given the heightened rhetorical prominence the World Health Organization has afforded to equity in the past half-century, it is important to better understand how equity has been referred to and its conceptual underpinning, which may have broader global implications.

**Eligibility criteria:**

Articles were included if they met inclusion criteria — chiefly the explicit discussion of the WHO’s concept of health equity, for example in terms of conceptualization and/or definitions. Articles which mentioned health equity in the context of WHO’s programs, policies, and so on, but did not discuss its conceptualization or definition were excluded.

**Sources of evidence:**

We focused on peer-reviewed literature by scanning Ovid MEDLINE and SCOPUS databases, and supplementing by hand-search.

**Results:**

Results demonstrate the WHO has held — and continues to hold — ambiguous, inadequate, and contradictory views of equity that are rooted in different theories of social justice.

**Conclusions:**

Moving forward, the WHO should revaluate its conceptualization of equity and normative position, and align its work with Amartya Sen’s Capabilities Approach, as it best encapsulates the broader views of the organization. Further empirical research is needed to assess the WHO interpretations and approaches to equity.

## Introduction

Since the late 1970s, equity in the context of health has become a central objective for the World Health Organization (WHO), largely attributed to the Alma-Ata Declaration of 1978, which emphasized the unacceptable nature of gross global health inequality and called for an acceptable level of health for all by the year 2000 [1]. A little over a decade after the declaration, the WHO commissioned a definition of inequity that has come to be widely cited globally: “differences which are *unnecessary* and *avoidable,* but in addition, are considered *unfair* and *unjust*” ([2], p. 5).

With the more recent Commission on the Social Determinants of Health (CSDH), convened by the WHO from 2005 to 08 to focus on equity [3], there is better recognition that social and economic inequality reduces social cohesion, unfairly distributes life chances, and results in inequalities in health outcomes [4]. Arguably, this has heightened the prominence of the social determinants of health and equity in the context of health.

Looking to the Whitehead [2] definition and others, a central difference between inequality and inequity is the moral imperative (unfair and unjust). Whereas inequality is a measured difference, inequity is a political concept with a moral commitment to social justice [5]. In other words, to determine if an inequality is an inequity, a moral judgment is required to determine what aspects of an inequality are unjust and unfair [5].

However, despite this distinction, there is little global consensus on the definitions of “health inequity,” “health inequalities,” or “health disparities” ([6], p. 167 [7];) and substantial differences remain in how health equity is defined and operationalized [8]. Notably, in international discourse and implementation, inequity and inequality are used interchangeably [9].

Evidently, even across shared terminology, discourses can vary drastically, as seen with health promotion discourses in WHO charters [10] and the aforementioned distinction between inequality and inequity. This is highly problematic, as differing underlying discourses (and potentially alignment with different theories and approaches) can yield different public policy implications for action [11]. Accordingly, different approaches can have implications in measurement and accountability [7]. Therefore, working to eliminate misunderstandings and move towards a shared understanding becomes increasingly important to bridge action.

Given that “the relative dominance of specific ontological, epistemological and praxiological stances in the global agenda for health equity are rarely discussed and often limited to rather linear accounts on the historical development of a specific agenda or a specific field of research” ([12], p. 3), an investigation into the WHO’s concept of equity is needed to better understand the root(s) of their stance(s). Accordingly, this study aims to map scholarly literature that critically examines the WHO’s conceptualization and interpretation of “equity” in the context of health (with an investigation into the WHO not restricted to one issue or division). This scoping review, to the best of our knowledge, is the first to review and examine the literature on how this global organization conceptualizes equity.

## Methods

### Literature search and search strategy

This scoping review, as described by Grant and Booth [13], is a preliminary review to assess the size, scope, nature, and extent of available literature. This type of review employs a systematic and comprehensive search process to ensure no citations are missed. However, in addition to traditional scoping reviews, this review seeks to include a conceptual analysis and critical insights, in addition to narrative commentary.

The research process was based on the PRISMA Extension for Scoping Reviews (PRISMA-ScR): Checklist and Explanation [14] and guided by Arksey & O’Malley’s [15] five procedural steps for scoping reviews. The scoping review protocol for this study was published in a peer-reviewed journal (Amri, Siddiqi, O’Campo, Enright, & Di Ruggiero, 2020). First, the research question was identified. Second, relevant literature was identified through a search of electronic literature databases (complemented by additional hand-searching). Third, independent iterative selection was undertaken by two reviewers, M.A. and G.J.-P., and final articles were also approved by A.S. and P.O. Fourth, charting pertinent data by M.A. independently. And lastly, key findings were collated using NVivo 12 and results summarized by M.A.

#### Electronic literature databases

The search was conducted from database inception to October 7, 2019, using two electronic literature databases: SCOPUS, an interdisciplinary database, and Ovid MEDLINE, a medical science database. These databases were selected through consultation with a research librarian, as they are believed to yield the most relevant and largest results.

In SCOPUS, the following search string was used to search in titles, abstracts, and by keywords:

“World Health Organization” AND “equit*” OR “inequit*” OR “equalit*” OR “inequalit*”, yielding 1875 citations.

Similarly, in Ovid MEDLINE, the following search string was used to search titles and abstracts:

“World Health Organization”.tw AND equit*.tw OR inequit*.tw OR equalit*.tw OR inequalit*.tw, yielding 739 citations.

#### Additional search procedures

In addition to these two databases, two influential papers were searched in Google Scholar to include papers that cited these two papers and contained “equity” in their title. From the 108 papers that cited “What does equity in health mean?” [16] on Google Scholar, 33 contained “equity” in the title.

From the 3176 papers that cited “The concepts and principles of equity and health” [17] (please note: this is the same text as Whitehead [2]), which contained 16 versions on Google Scholar, 500 contained “equity” in the title.

#### Elimination of duplicates

After removing duplicates from the identified papers (both using EndNote X9 and manual elimination), this resulted in 2538 non-duplicate citations. An additional 20 citations were included through hand-searching, largely drawn from other articles’ citations, yielding 2558 articles for which the critical review of the literature was undertaken.

### Eligibility

#### Inclusion criteria

Drawing on the 2558 citations, article titles and abstracts were first reviewed for relevance using a priori inclusion and exclusion criteria by two independent reviewers, M.A. and G.J.-P., and all potentially relevant articles were read in full to determine and ensure alignment with these criteria. Articles were included if they met inclusion criteria — chiefly the explicit discussion of the WHO’s concept of health equity, for example in terms of conceptualization and/or definitions. Articles which mentioned health equity in the context of WHO’s programs, policies, and so on, but did not discuss its conceptualization or definition were excluded. As such, the seminal Whitehead [2] paper which was used in the search strategy was excluded from the review. Final papers included in the scoping review were also reviewed and approved by A.S. and P.O. to ensure suitability with inclusion criteria.

Please note, this study intentionally did not restrict the selection of articles by type of paper (commentaries, editorials, literature reviews, analysis papers, etc.) nor by year, to aid in understanding if and how work in this field and broader understanding changed over time, particularly with the development of the CSDH. Only papers available in English were included.

#### Exclusion criteria

Studies were excluded if they did not meet the inclusion criteria identified above. Some papers that were excluded include those that:

1. State the definition of equity without any further analysis or discussion. This includes those where the WHO’s definition of equity is drawn on as a side-point or casually referred to without additional interrogation and those that discuss equity broadly and are not specific to the WHO. For example, papers that highlight the need for action to tackle health inequalities (but no critique or discussion around the meaning of equity). Or, those which reiterate what the WHO is already doing about equity (without discussing the underlying discourses).

(2) Solely focus on specific inequities (e.g. inequity of genetic testing) instead of a discussion on equity, equity in health, or health equity more broadly. This also includes articles that discuss a specific health issue/disease/condition and concludes that there are implications for equity (e.g. inequitable distribution of health care professionals, asthma, tuberculosis).

(3) Solely focus on the measurement of inequity or inequality, some of which include: epidemiological/statistical, case-control, cross-sectional studies, the study of one country/population group, etc. However, papers that drew on a discussion of measurement to delve into discourse, theory, normative positions, etc. were included (e.g. “A Problem with the Individual Approach in the WHO Health Inequality Measurement” [18]).

(4) Were unavailable in English — due to resource restrictions.

## Results

After applying the inclusion and exclusion criteria to the initial screen of titles and abstracts, 2325 articles were excluded (including eight abstracts that were unavailable in English and 30 abstract texts that were either unavailable or unable to access). After conducting a full-text screening of the 233 abstracts that were identified for full-text review, 212 articles were excluded (including 42 full texts that were unavailable in English and five full texts that were either unavailable or unable to access). During the data extraction process, two citations were excluded due to repetition of full text in another article. There were 23 articles included in the final review that met the inclusion criteria (see Table 1).

**Table 1:** Papers collated from scoping review (*n* = 23)

	Author(s)	Author’s/s’ institution(s)	Title	Type of publication	Place published	WHO’s definition or approach to equity	Conclusions
**1**	Alleyne [19]	The Pan American Health Organization (PAHO)	Equity and the goal of Health for All	Speech	Pan American Journal of Public Health	Equity sought through Health for All focused on distributive justice/an egalitarian approach, which may not be beneficial.	What is needed is attention to the unequal distribution of the determinants of health inequalities and concept of equity in health that cannot be separate from equity in broader human development (aligned with Amartya Sen’s Capabilities Approach).
**2**	Asada [20]	Dalhousie University	Is Health Inequality Across Individuals of Moral Concern?	Journal article	Health Care Analysis	The Whitehead definition makes the distinction that health inequities have a moral judgment (i.e. not all inequalities are inequities), while leaving the exact meaning of health inequity open. The WHO implies that health should be prioritized over other goods through seeking to measure health inequalities across individuals versus groups, which can be considered to be specific egalitarianism or a direct approach.	Health inequity is of moral concern (as the WHO purports) due to the role of health equity in justice and health inequality being an indicator for broader societal injustice. Ultimately, how health inequalities are measured implies certain moral perspectives. As such, policy makers should determine which perspective is suitable. For example, while the Capabilities Approach is rooted in equality and justice, it focuses on those below a minimum versus the whole distribution of health.
**3**	Asada and Hedemann [18]	University of Wisconsin-Madison	A Problem with the Individual Approach in the WHO Health Inequality Measurement	Journal article	International Journal for Equity in Health	By measuring inequalities between individuals, as opposed to groups, the WHO’s health inequality measure is not value-free and represents an “expansive view of justice” that sees inequities as those amenable to intervention.	The conceptual underpinning of the WHO’s health inequality measurement/index needs to be re-evaluated.
**4**	Bambas and Casas [21]	The Pan American Health Organization (PAHO)	Assessing Equity in Health: Conceptual Criteria	Report chapter	Equity & Health: Views from the Pan American Sanitary Bureau	Whitehead’s definition is a robust concept of equity but has controversial parameters.	Equity should be considered on a spectrum from “misfortune” to “inequity”.
**5**	Blakely [22]	University of Otago	Iconography and Commission on the Social Determinants of Health (and health inequity)	Journal article	Journal of Epidemiology and Community Health	While the CSDH final report focuses on power, sexism, and discrimination, the mention of “power” and “racism” is removed in the final diagram of the social determinants of health (and used as a model of health inequalities), despite the CSDH including “power”, “class”, “racism”, and “discrimination” in a prior working diagram.	The CSDH report, which is rooted in values of fairness and altruism, provides an authoritative account of health inequalities, both in terms of knowledge to-date and what actions need to be taken, and has the potential to be a landmark report.
**6**	Bommier and Stecklov [23]	Institut National d’etudes Démographiques & Hebrew University of Jerusalem	Defining health inequality: why Rawls succeeds where social welfare theory fails	Journal article	Journal of Health Economics	The WHO, through the Global Strategy for Health for All resolution (WHA32.30), WHO Health for All, and Whitehead definition, implicitly indicates that a fair health distribution entails reducing avoidable health differences, not equal health status for all individuals (given differing individual endowments).	The social welfare approach is inconsistent with a social justice approach’s notion of a just or fair distribution. Authors propose an alternative approach which they state seemingly aligns with the WHO’s approach to equity and Rawls’s ethical principles.
**7**	Borde and Hernández [12]	Universidad Nacional de Colombia	Revisiting the social determinants of health agenda from the global South	Journal article	Global Public Health	The WHO’s approach to equity has been driven by the CSDH approach, which is rooted in Anglo-Saxon European Social Medicine, as opposed to other approaches, such as the Latin American Social Medicine and Collective Health approach. Authors also note the WHO’s approach aligns with Amartya Sen’s notion of health as a “special good”.	In seeking to address equity, the WHO focuses on technical solutions, seeks win-win solutions for governance, and renders politics apolitical, but should focus on the social determinants of health inequities, or causes of causes (i.e. unequal power relations or capitalism). As such, the principles and foundations (ethical, political, etc.) need to be clarified and engage with ‘invisibilized’ approaches.
**8**	Braveman [6]	University of California (San Francisco)	Health Disparities and Health Equity: Concepts and Measurement	Journal article	Annual Review of Public Health	Expresses the acceptance of Whitehead’s definition but indicates the context in which it was created emphasizes inequalities between those of differing socioeconomic status, and rarely gender and ethnicity.	Need for a new definition that specifies the importance of social position, types of comparisons to be made across and within groups, and has explicit criteria. This can be done by drawing on the right to health (i.e. using the most privileged as a reference group).
**9**	Braveman and Gruskin [7]	University of California (San Francisco); Harvard University	Defining equity in health	Journal article	Journal of Epidemiology and Community Health	The Whitehead definition has raised awareness of the meaning of health inequities, but ‘avoidability’ should not define health inequities because: (i) avoidability is implied through being unfair and unjust, (ii) given that many inequities require structural changes, avoidability may hinder action on inequities, and (iii) avoidability is unclear about who it is avoidable by. In addition, the WHO’s argument for measuring inequalities by individuals rather than groups does not afford consideration of health equity and does not reflect values of fairness or justice.	Clarity is needed to ascertain how definitions of health equity align with paradigms and determine what the practical implications of such definitions are.
**10**	Daniels, Kennedy [24]	Harvard School of Public Health; Cambridge, MA; & Harvard School of Public Health	Health and Inequality, or, Why Justice is Good for Our Health	Book chapter	Public Health, Ethics, and Equity	The Whitehead definition, while useful, is only beneficial if agreement can be reached on what constitutes avoidable and unfair. Broadly, the WHO’s efforts are aimed at remedying true inequities.	Drawing on Rawls theory of justice can be beneficial in determining which health inequalities are inequities. Policy should be intersectoral to address social conditions and provide equality of opportunity, capabilities, or positive freedom (rather than strictly health*care*).
**11**	Gwatkin [25]	The World Bank	Health inequalities and the health of the poor: What do we know? What can we do?	Journal article	Bulletin of the World Health Organization	While the WHO approach to equity is a normative one largely focused on reducing inequalities in social position, there are also aspects focused on improving the health of the impoverished.	Need for the development of relevant equity-oriented health objectives (e.g. % reduction of disparities and improving conditions among poorer groups) (which are rare compared to goals expressed as improving or reducing rates in specific indicators across a population) and enhanced information collection and tracking.
**12**	Hashimoto and Kawakami [26]	University of Tokyo	Health equity	Book chapter	Oxford Textbook of Public Mental Health	Observes that PAHO interprets inequity as a difference in the opportunity to achieve health (i.e. equity equates to equal opportunity to achieve health).	Health equity should not be focused solely on the equal distribution of health status, but also functionings and freedoms, as expressed in the Capabilities Approach.
**13**	Hausman, Asada [27]	University of Wisconsin-Madison	Health Inequalities and Why They Matter	Journal article	Health Care Analysis	The WHO approach to measuring inequalities is not value-free and only measures group differences at the national level, missing intra-group inequalities.	Measuring of inequality should be done by assessing health outcomes at various life stages and in relation to other factors, rather than studying the distribution of health expectancies.
**14**	Linares-Péreza and López-Arellano [28]	Universidad del Valle de Guatemala & Universidad Autónoma Metropolitana Unidad Xochimilco	Health Equity: Conceptual Models, Essential Aspects and the Perspective of Collective Health	Journal article	Social Medicine	The WHO attempts to approach equity through consideration of the health needs of society, instead of just social privilege.	A comprehensive vision of health equity that considers both the field of health (equity in outcomes, access, and care) and environment (equity in social, economic, cultural, and political determinants and daily living conditions) is needed.
**15**	Marmot and Friel [29]	University College London; (Friel’s affiliation not listed)	Global health equity: evidence for action on the social determinant of health	Journal article	Journal of Epidemiology and Community Health	The CSDH was guided by the underlying value that health inequities are a matter of social justice.	Both reducing societal inequities (i.e. reducing the gap) and improving the health of the whole of society (i.e. improving everyone’s health) are important.
**16**	Mooney [16]	University of Copenhagen	What does equity in health mean?	Journal article	World Health Statistics Quarterly	There is a lack of clarity behind the WHO’s Health for All objective(s).	Need to clarify concepts of equity in practical terms and be more explicit about policy goals for health equity.
**17**	Pappas and Moss [30]	Johns Hopkins University	Health for All in the Twenty-First Century, World Health Organization renewal, and equity in health: a commentary	Journal article	International Health Agencies	The WHO’s approach to equity in Health for All in the Twenty-first Century is divided along the historically-rooted lines of prevention and care, yielding philosophical and pragmatic implications. The WHO conceptualizes equity in an implicit way, yielding a lack of explicit policy actions.	The WHO needs to clarify how policy can be used to achieve health equity, perhaps by drawing on the rights-based approach. The WHO should lead in ensuring validity, reliability, and timeliness of data from around the world, and ensure progress is measured through specific, qualifiable, and culturally-appropriate targets (e.g. develop socioeconomic measures that consider culture and economic development).
**18**	Ridde, Guichard [31]	University of Montreal; Institut National de Prévention et d’Éducation pour la Santé; & Université Catholique de Louvain	Social inequalities in health from Ottawa to Vancouver: action for fair equality of opportunity	Journal article	Promotion & Education	Equity has moved towards achieving the best possible state of health for individuals instead of ultimate equality. As well, the Ottawa Charter for Health Promotion is founded on the principle of equity based in distributive justice.	*Fair* equality of opportunity proposed by Rawls (i.e. not disadvantaging any individual in seeking to reach their full health potential), should be advocated for (opposed to equality of opportunity). There is a need to evaluate action on health equity.
**19**	Sadana and Blas [32]	WHO headquarters; WHO Regional Office for Europe	What Can Public Health Programs Do to Improve Health Equity?	Journal article	Public Health Reports	Inequity reflects a value judgement and the CSDH determined that about 75% of health inequalities can be considered unfair and potentially unavoidable. In addition, the WHO Priority Public Health Conditions Knowledge Network determined that programs studied largely focused on treatment and partially on vulnerabilities of groups, with the exception of violence & injury prevention and tobacco, which addressed the upstream social determinants of health.	Upstream action is needed and there is an opportunity for program areas of the WHO to intervene on shared social determinants of health. And regardless of where the determination is made of what constitutes an inequality vs. inequity, action needs to be taken on reducing unfair health disparities.
**20**	Shiell [33]	University of Calgary	Still waiting for the great leap forward	Journal article	Health Economics, Policy and Law	The CSDH was only concerned with reducing inequity (particularly inequalities in health between countries and groups within countries), and not concerned with prioritizing certain groups (with the exception of prioritizing for efficiency) or competing ethical considerations (the authors imply the latter two are also needed). The CSDH also prioritizes social justice (as opposed to individual liberties).	Economic evaluation evidence is needed for interventions that address the social determinants of health, as it can help reframe social justice and inequalities to combat ideas of constrained individual liberties in political debates. However, economic evidence is also not sufficient, as interventions targeting the upstream determinants are needed.
**21**	Smith [34]	University of Toronto	Health Equity in Public Health: Clarifying our Commitment	Journal article	Public Health Ethics	The Whitehead definition of health equity is inadequate normatively (i.e. how are ‘unnecessary’ and ‘avoidable’ differences constituted?), and as a result, may result in inconsistent or conflicting action and potentially the creation of inequities.	Clarifying health equity (particularly around fit with social justice and foundational concept of justice) will afford alignment of goals in policy and practice.
**22**	Smith and Normand [35]	Economic and Social Research Institute (Ireland); Trinity College	Equity in health care: the Irish perspective	Journal article	Health Economics, Policy and Law	Determining whether or not an inequality is unfair rests on degree of choice, which is open to criticism. While it is difficult to discern exactly which theoretical perspective the Whitehead definition is based in, the definition is unlikely to be aligned with a libertarian philosophical perspective and resonates with Rawls’ theory of social justice (this definition was adapted for use in Ireland).	Health equity is a difficult concept to define and has resulted in conflicting approaches. Lessons from Ireland can work to inform other jurisdictions.
**23**	Wilson [36]	University College London	Health inequities	Book chapter	Public Health Ethics: Key Concepts and Issues in Policy and Practice	The Whitehead definition’s inclusion of “unnecessary”, “avoidable”, and “unfair” can be eliminated to simply remain “unjust inequality”. However, determining which inequalities are unjust is a difficult and complex task.	Individuals who are worst off should be prioritized and their health (e.g. over socio-economic status) should be concentrated on (i.e. favouring a pluralistic theory of justice over a monistic one).

Excluding authors based at the WHO/PAHO and World Bank as listed in their stated affiliation (*n* = 4/23; 17.4%), only two papers (*n* = 2/19; 10.5%) had authorship from a non-high-income economy (as determined by the World Bank [37]) -- and in fact, both of these were from upper-middle-income economies (one paper authored by a scholar in Colombia [12] and another authored by scholars in Guatemala and Mexico [28]).

Looking to the authors’ affiliations at the time of publication, the majority has authorship at a university (*n* = 19/23; 82.6%). Three were based at the WHO/PAHO (*n* = 3/23; 13%) and one was authored by an individual at the World Bank (n = 1/23; 4.3%).

Reviewing the timeline of publications included in this scoping review, only 13 articles were published from 2005 and onwards (*n* = 13/23; 56.5%) and only seven were published from 2009 to present (*n* = 7/23; 30.4%) (which is noteworthy, given that the CSDH convened from 2005 to 2008).

**Fig. 1:**
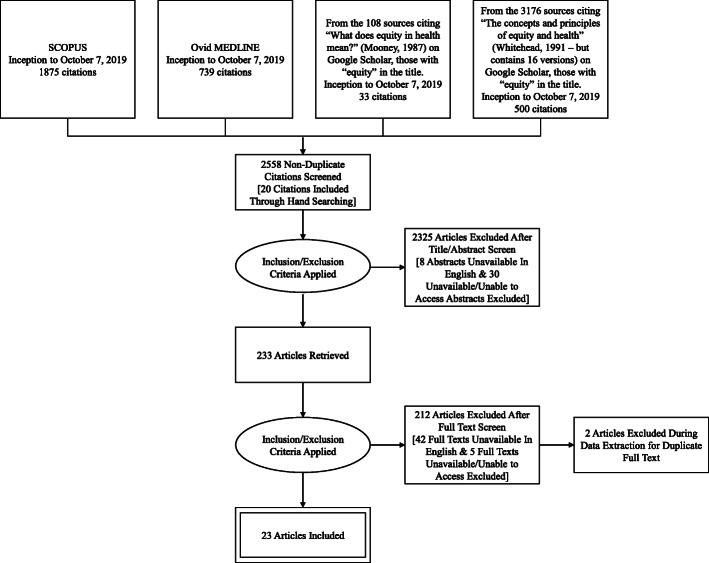
Search strategy based on the PRISMA Extension for Scoping Reviews (PRISMA-ScR): Checklist and Explanation [14]

Figure 1 provides an overview of the search strategy employed, detailing how papers were identified, screened, deemed eligible, and included.

From the final 23 articles, data on key study descriptors were extracted [e.g. author(s) and year of publication, author’s/s’ institution(s), title, type of publication, place published, reflections on the WHO’s approach to equity, and conclusion(s)].

### Critical review of the literature

This scoping review of the academic literature is designed to yield insights on the depth of interrogation of the WHO’s discussion of equity. It aims to identify key discussions in the field and identify any gaps, if they exist, so that they can be addressed to improve global health policy.

As such, in addition to article data abstracted in Table 1, deeper information through reading and interpreting, such as comparing, analyzing, and synthesizing critiques of the WHO’s approach (es), were focused on (e.g. in discussion around the definition by Whitehead [2] and any perceived normative or theoretical positions of the WHO).

## Discussion

From the academic papers meeting the inclusion criteria of this study, as outlined in Table 1, key ideas were illuminated that will be discussed in this section.

### Widespread use of Whitehead definition

A large majority of papers referred to Whitehead’s formative definition [2] commissioned by the WHO, which was expressed to be defined the same way by the Pan American Health Organization (PAHO) [28].

While originally intended to raise awareness and debate among a wider general audience [7, 38], this definition provides an understanding that inequalities produced by the social system are inequities, while inequalities produced by nature are not inequities [18]. In other words, inequities are not only unnecessary and avoidable (inequality), but are unfair and unjust (inequity). Consequentially, the recognition and acceptance of inequities being *unjust* in Whitehead’s definition has a moral undertone because a value judgement must be made to determine what is an inequity versus inequality [31], demonstrating the close link between equity and social justice [25].

The widespread acceptance of Whitehead’s definition was clear, whether this was done by explicitly referring to Whitehead and quoting the definition (e.g. [23, 26]) and expressing the widespread acceptance of the definition itself (e.g. [25, 34]), or indirectly, by recognizing the “general agreement” that inequalities are unjust (e.g. [19]). This Whitehead definition has been recognized as having the key strengths of being accessible, concise, intuitive, and easily communicated [6]. Despite several definitions of equity in the literature, Whitehead’s has been widely taken up [6], demonstrating the role this definition had in providing a widely accepted baseline of understanding for inequity in health or health inequities.

### WHO’s approach is ambiguous and inadequate

Prior to Whitehead’s definition, Mooney [16] raised concerns that the WHO’s Health for All initiative was unclear about equity. While Whitehead’s definition of equity has been widely accepted since its development in 1990, it has become apparent through this review that it is *highly* ambiguous and inadequate.

Despite inequities being understood to be “not only unnecessary and avoidable, but in addition, are considered unfair and unjust” [2], it is immensely difficult to determine what is in fact, “unnecessary and unfair vis-à-vis what is inevitable and unavoidable” [21]. For example, ‘avoidability’ has several subcategories: technical, financial, and moral [21]. Similarly, Wilson [36] has expressed that the addition of “unnecessary and avoidable” is unhelpful and can be eliminated to simply remain “unjust inequality”. This was also argued by Braveman and Gruskin [7] who expressed that ‘avoidability’ should not define health inequities because: (i) avoidability is implied through being unfair and unjust, (ii) given that many inequities require structural changes, avoidability may hinder action on inequities, and (iii) avoidability is unclear about who it is avoidable by. Evidently, the Whitehead definition, while useful, is only beneficial if agreement can be reached on what constitutes avoidable and unfair [24], and determining which inequalities are unjust is a difficult and complex task [36]. As such, it leaves the exact meaning of health inequity open [20]. Similarly, these terms are open to varying interpretations which can be problematic [6, 19, 34]. For example, determining whether or not an inequality is unfair rests on the degree of choice [35] or the idea that inequalities with a social versus natural cause are amenable to intervention [36], both of which have been criticized.

Further, the WHO’s approach to inequities (measuring inequalities between individuals as opposed to groups) may miss inequalities that most would consider to be injustices, and therefore, should be considered inequities [18], demonstrating it is not only ambiguous but also inadequate. On the contrary, Sadana and Blas [32] point out that regardless of where the determination is made of what constitutes an inequality vs. inequity, action needs to be taken to reduce unfair health disparities.

In addition, ambiguities in the WHO’s approach to equity are present on a superficial level. Ridde, Guichard [31] draw attention to the translations of the Ottawa Charter, noting the French version refers to “equality in health”, whereas the English version refers to “equity in health”.^1^ Evidently, not enough attention has been given to studying why an inequality may be unfair and the implications of this characterization are [21]. And ultimately, ambiguities present in the WHO’s approach to equity were fraught with key contradictions, as discussed below.

### Key contradictions in the WHO’s approaches

*“[..] if an idea has an essential ambiguity, a precise formulation of that idea must try to capture that ambiguity rather than lose it”* [39]*.*

The WHO’s differing approaches to equity present three key contradictions that the literature highlighted, which centred around: (1) individual versus group measurement of health inequalities, (2) striving for a baseline level of health for all or striving for a baseline level of health for all AND reducing inequality, and (3) focus on socioeconomic status versus considering various social determinants of health.

#### Individual versus group measurement of health inequalities

The WHO has employed an approach centred on measuring differences across individuals at times, while measuring differences between groups in other instances. This is most evident through Braveman’s contrasting of the WHO’s 1995–1998 initiative on Equity in Health and Health Care, which specifically indicated equity as minimizing disparities *between groups* (with mention of those who have different levels of power, wealth, prestige), with researchers at the WHO from 1998 to 2003 who emphasized differences *between individuals* and *not* groups [6].

##### Individual measurement

Asada [20] points to researchers at the WHO which proposed to measure inequalities across individuals rather than groups through the *World Health Report 2000* due to methodological (e.g. ease of international comparisons) and moral reasons (i.e. due to health being special, the role of health equity in justice, and health inequality being an indicator for broader societal injustice). Through undertaking measurement of inequalities across individuals, Asada [20] infers that the WHO implies that health should be prioritized over other goods, which can be considered to be specific egalitarianism or a direct approach.

However, the measurement of health differences at the individual-level undertaken by the WHO does not allow for a determination of what differences are, in fact, determined by nature (inequality) versus socially-determined (inequity) [18]. In other words, the WHO’s argument for measuring inequalities by individuals rather than groups does not afford consideration of health equity and does not reflect values of fairness or justice [7]. As a result, Asada and Hedemann [18] hold the theory that the WHO does not use social factors to qualify inequalities as inequities, but rather, whether or not causes of differences are *susceptible to human intervention*. Asada and Hedemann [18] refer to instances in WHO texts that support this view, providing two examples to illustrate their point. First, inequalities caused by genetic makeup are considered inequities by the WHO given that genetic manipulation in the “era of the human genome project” is possible, despite these not being socially-determined [18]. And second, they give a hypothetical example where systematic discrimination in a state may result in health inequalities, and purport that the use of the WHO’s measure of inequality at the national level these differences would not be apparent and would determine it is an inequality rather than inequity [18].

##### Group measurement

In seeking to scientifically measure inequality, there is a decision made incorporating normative or causal assumptions in considering which questions are of moral importance [18]. In other words, while some measures are value-free (inequality), how we determine what is important to look at and address (inequity), has a moral component; with the analogy of measuring the height of trees given – where measurement data is unimportant until it is compared, ranked, and aspects to address are identified [18]. Because the concept of equity implicitly contains the issue of measurement [30], there appears to be a normative position that the WHO holds (i.e. what becomes worthy of our moral attention), despite claiming to be value-free. For example, through the WHO’s measurement of group differences at the national level, intra-group inequalities are missed [27]. This is not to say that it is unscientific, but rather that there are embedded values in how this science is conducted.

#### Striving for a baseline level of health for all versus striving for a baseline level of health for all AND reducing inequality

##### Striving for a baseline level of health for all

It is unclear as to whether or not the WHO approaches equity in health as envisioning individuals as having a baseline level of health or not. Mooney [16] provided the example of the European Strategy for Health for All, which Mooney claims the target seems to be equal health, raising questions around how this level of health is determined. This was supported by Gwatkin [25], who notes the WHO has also mentioned the need for a basic minimum level for all individuals across the population, and also raised by Alleyne [19], who explains Health for All forced a consideration of what was truly meant by “All” and implied distributive justice and a minimum level of health. He also states that equity in health aligned with the egalitarian approach and that he “confess [es] to my bias in this direction” [19] — which is noteworthy as he was a director of the PAHO.

Ridde, Guichard [31] also support this claim, as they perceive the 1986 Ottawa Charter for Health Promotion to be based in values of distributive justice given the social construction of inequalities (with distributive justice either denoting equal material goods for all or diverging when it allows for those worse off to benefit more than in the former scenario [40]). This is further supported by Hashimoto and Kawakami [26] who observe that the PAHO interprets inequity as a difference in the opportunity or means to achieve health (i.e. equity equates to equal opportunity to achieve health). Through the PAHO’s approach, the notion that equity is perceived by the WHO as one in which individuals have a baseline level of health is clear and yields a clear approach to combatting inequity.

##### Striving for a baseline level of health for all AND reducing inequality

Mooney [16] points to a contrast between the WHO’s European strategy for health for all, which focuses on raising up those who are not as privileged, and the WHO’s Global Strategy for Health for All, which focuses on reducing inequality between people’s health status.

Gwatkin [25] also notes this inconsistency by noting how the definition of health equity developed by Whitehead [2], which called for improving the health of the disadvantaged, was developed alongside another definition that focused on reducing differences between groups. Similarly, Gwatkin [25] observes that the WHO’s 1996 health equity document gives importance to differences between the poor and rich, rather than just focusing on enhancing the health of those with lower socioeconomic status.

Looking to more recent work of the WHO, Shiell [33] also makes a similar observation through looking at the work of the CSDH. Shiell [33] notes that the CSDH was only concerned with reducing inequity (particularly inequalities in health between countries and groups within countries), and not concerned with prioritizing certain groups (with the exception of prioritizing for efficiency) — despite Marmot and Friel [29] expressing that the work of the CSDH was focused on both reducing societal inequities (i.e. reducing the gap) and improving the health of the whole of society (i.e. improving everyone’s health).

While the WHO approach to equity is a normative one *focused* on reducing inequalities in social position, one may argue equity should not only be focused on reducing disparities (e.g. can focus on improving the health of the impoverished) [25]. Further, Mooney [16] draws attention to the goal of “drastically reduced” gross inequality in health expressed in the WHO’s Global Strategy for Health for All, to explain that while this is still unclear, it is more feasible than the European Strategy for Health for All goal of achieving equal health. Bommier and Stecklov [23] also note that the wider literature points to an understanding that a fair distribution of health does not equate to equal health status for all, but rather around reducing or eliminating avoidable differences, which they state is implicit in the work of the WHO through the Global Strategy for Health for All resolution (WHA32.30), WHO Health for All, and Whitehead definition. Irrespective, the differing approaches to health equity by the WHO becomes apparent.

#### Focus on socioeconomic status versus considering various facets of inequity

##### Focus on socioeconomic status

As Braveman [6] highlights, while Whitehead does not explicitly mention “disadvantage”, her notions and examples of “avoidability,” “injustice,” and “unfairness” are intended to demonstrate inequalities in socioeconomic status. This is supported by equity in health focusing on reducing disparities among those with different levels of social advantage and privilege, with the examples of differing levels of power, wealth, and privilege, given by the 1995–98 WHO initiative on Equity in Health and Health Care [6]. Similar language is employed by both the WHO’s European Strategy for Health for All, which strives to allow “disadvantaged nations and groups [ …] catch up” [16] and was present in the 1984 targets for the WHO Regional Office for Europe [25].

Interestingly, the European context in which the Whitehead [2] definition was created emphasizes inequalities between those of differing socioeconomic status, and rarely gender and ethnicity [6]. In the same vein, Pappas and Moss [30] called for the WHO to develop socioeconomic measures that consider culture and economic development, highlighting the limited consideration for each individual’s unique situation.

##### Considering various social determinants of health

However, despite Mooney’s claims, Linares-Péreza and López-Arellano [28] make an astute observation that Whitehead [2] indicated that not all individuals should utilize the same degree of resources, nor the same level of health, and instead, each individual’s needs should be considered.

Arguably, the WHO attempts to approach equity through consideration of the health needs of different societies (i.e. through differences in ethnicity, religion, socioeconomic status, gender, geography, and age), instead of social privilege [28]. This is supported by the assertion made by Daniels, Kennedy [24], that the WHO’s efforts are aimed at remedying “true inequities” (i.e. health inequalities among socioeconomic status and racial/ethnic groups). This approach of considering various facets of inequity is clear through Hashimoto & Kawakami’s [26] discussion of equity in the context of health, where they discuss alignment between the WHO’s International Classification of Functioning, Disability and Health and the Capabilities Approach, developed by Amartya Sen, as it treats inclusion as a functioning/key aspect. Braveman [6] also notes researchers at the WHO from 1998 to 2003 argued against focusing on inequality and inequity of socioeconomic status, but instead, defining inequity as an avoidable difference between individuals without social grouping.

Further, through looking at the work of the CSDH, Sadana and Blas [32] point to the use of the CSDH’s conceptual framework in demonstrating how various health determinants contribute to inequities. In particular, pointing out that the ninth knowledge network focused on priority public health conditions largely focused on treatment and only partially on vulnerabilities of groups, except for violence & injury prevention and tobacco, which addressed the upstream social determinants of health [32].

When discussing equity in the context of health*care*, Dahlgren and Whitehead [38] indicate “fair arrangements that allow equal geographic, economic and cultural access to available services for all in equal care of need”. This statement points to an expanded vision of equity from one focused on wealth, to one which considers these three dimensions.

### The WHO’s apparent values and conceptual underpinning

It is important to note that how inequities are “conceived, conceptualised, researched and proposed to be transformed, and consequently mobilise different political, social and economic agendas” [12]. For example, different definitions of health equity align with different paradigms, which have different practical implications [7]. While the unfair and avoidable aspect of differences in health are becoming more apparent and greatly acknowledged [41], the WHO’s discussion and approach to equity is evidently quite ambiguous, inadequate, and contradictory.

The WHO approach to inequity aims to be scientific, but it is not value-free [27] — despite the WHO’s claims its measurement of health inequality is value-free [18] — illustrated through the explicit mention of the CSDH being guided by the underlying value that health inequities are a matter of social justice [29]. The WHO is not “wedded to any specific empirical theory of inequality” [27], but its normative position “can be interpreted as a quite expansive view of justice” [18]. Through the adaption of the Whitehead definition to the Irish national health strategy, Smith and Normand [35] reiterate this sentiment, by expressing that while it is difficult to discern exactly which theoretical perspective the definition is based in, it is unlikely to be aligned with a libertarian philosophical perspective and resonates with Rawls’ theory of social justice. While the Whitehead definition of health equity is inadequate normatively, which may result in inconsistent or conflicting action (and potentially the creation of inequities) [34], it is not just merely the Whitehead [2] definition that has an ambiguous or contradictory theoretical underpinning.

Through striving for a baseline level of health for all (as discussed above), the WHO’s approach aligns with notions of strict egalitarianism, which seeks to ensure the same level of goods and services for all [40], or specific egalitarianism or a direct approach [20]. Similarly, through the WHO’s enhanced focus on eliminating differences due to socioeconomic status, this aligns with Rawls’ approach [42], which focuses on “primary goods” (such as income and wealth). His Difference Principle allows inequality only to raise those most disadvantaged in society [40], which is believed to align with the WHO’s approach to equity [23]. While these approaches have merit, they fail to consider the diversity among individuals (i.e. two individuals with the same level of primary goods may have different freedoms to achieve their potentially unique perceptions of a good outcome) [39].

Further, by the WHO’s aim to consider individuals’ unique needs, as noted by Linares-Péreza and López-Arellano [28] (as discussed above), the WHO’s approach aligns well with the Capabilities Approach developed by Amartya Sen. Sen’s Capabilities Approach posits that a person’s capability to achieve “functionings” that they value, ranging from basic needs (e.g. avoiding morbidity) to complex achievements (e.g. having self-respect) — provides a valuable approach to assessing (in) equality [39]. The approach considers how choices are made, rather than simply welfare [26] and aligns with the idea that the distribution of health has moral significance, which was expressed by WHO researchers [20]. And in fact, Sen not only served as a Commissioner on the 2005–2008 CSDH, but his thinking is regarded as being influential in setting up the CSDH [43].

As it may now be apparent, Sen’s approach seemingly best aligns with the views of WHO key figures (e.g. Alleyne [44] and Dahlgren and Whitehead [38]^2^) — who indicate geographic, economic, and cultural factors, among others, should be considered in striving for health equity. Given that there are various philosophical perspectives on health equity, determining a perspective for which to align policy with is recommended (i.e. not only for aligning sought-out policy outcomes but also because health inequality data can be interpreted in different ways) [20]. As such, grounding and aligning the WHO’s approach in the Capabilities Approach, as opposed to one unitary theory of social justice, may aid in clarifying a path forward. This may be aided by discussions around fairness and equality, which can help clarify what the WHO means by equity [30].

If the WHO were to align their approaches to equity with that of Sen’s Capabilities Approach, it may aid in various pursuits. For example, Pappas and Moss [30] called for the WHO to ensure progress is measured through specific, qualifiable, and culturally-appropriate targets (e.g. develop socioeconomic measures that consider cultural and economic development). By approaching the measurements of systematic differences with Sen’s Capabilities Approach as a lens, this call could potentially be even better addressed. Similarly, neither the Ottawa Charter of 1986 [31] nor the WHO’s Health for All in the Twenty-first Century policy [30], clarify how equity will be addressed in explicit ways or describe clear actions to achieve equity. Through broadening the view to equity to one that considers an individual’s broader society, considerations for enhancing equity can more easily (and should) focus on political contexts (including oppression and exploitation), as called for by Borde and Hernández [12]. This could potentially aid in filling the gap identified by Ridde, Guichard [31] that there is a “permanent invisibility” of inequalities in health politically and by Pappas and Moss [30] to provide the much needed clarity on how policy can be used to achieve health equity.

In fact, Asada and Hedemann [18] concluded that “if the World Health Organization’s health inequality measure is to be interpreted meaningfully in a policy context, its conceptual underpinning must be re-evaluated”. For example, Asada [20] asserts that in terms of measurement, the Capabilities Approach focuses on those below a minimum versus the whole distribution of health, demonstrating how theories of justice can have direct implications on policy and practice. Further to this, Borde and Hernández [12] highlight that the WHO focuses on technical solutions, seeks win-win solutions for governance, and renders politics apolitical. Instead, the WHO should clarify its principles and foundations (ethical, political, etc.) and focus on the social determinants of health inequities, or causes of causes (i.e. unequal power relations or capitalism) [12]. This is supported by an observation made by Blakely [22], that while the CSDH final report focuses on power, sexism, and discrimination, the mention of “power” and “racism” is removed in the final diagram of the social determinants of health (and used as a model of health inequalities), despite the CSDH including “power”, “class”, “racism”, and “discrimination” in a prior working diagram.

### Potential promise of the rights-based approach

In the review of these papers, the role of the human rights-based approach in striving for equity was raised by a few authors. This approach emphasizes legislative and legalistic actions (e.g. through targeting constitutions, laws, and court action) [30]. In doing so, equity moves into the domain of law and the responsibility of government, rather than strictly an act of goodwill [12].

Pappas and Moss [30] note there may be an opportunity for the WHO to draw on the rights-based approach, like the United Nations Children’s Fund (UNICEF), to clarify how policy can achieve health equity and guide its actions. With Sadana and Blas [32] explicitly pointing to the alignment of a social determinants of health approach with the promotion of human rights (such as around healthcare, education, safe water, and a decent standard of living). However, Mooney [16] explains that while the WHO Global Strategy for Health for All asserts that “health is a fundamental human right”, parameters to a right to health are unclear, as opposed to healthcare.

### Limitations

While this scoping review employed a systematic and rigorous search strategy, there were two limitations to the study.

First, because only articles available in English were included in the study due to resource constraints, only English-speaking voices, perspectives, and analysis were collated. Particularly because the WHO is a global organization, and with equity being rooted in different traditions of social justice across the world [30], additional efforts are needed to review papers published in other languages.

Second, because some articles were written by individual authors (e.g. Dr. George Alleyne) who were employed at the WHO, this blurs the lines of what constitutes the organization’s position versus individual authors’ views. However, careful consideration was paid to the purpose of each article and the author’s voice [e.g. Alleyne explicitly presented his views, e.g. “I confess to my bias in this direction” [19], as opposed to the views of the broader organization].

## Conclusions

As Dahlgren and Whitehead [38] indicate, the original document containing Whitehead’s formative definition of equity was intended to raise awareness and debate among a wider general audience. Now that this goal has largely been accomplished, the WHO needs to clarify not only what equity in the context of health means to the organization, but what values and theory of justice underpin this. Sen’s Capabilities Approach has been expressed to represent the values of the organization by prominent players but does not represent the organization’s policy and program actions.

In assessing the literature that this scoping review analyzed, the need for more discussion among scholars from the global south and representation of these voices is glaring. Given different traditions of social justice across the world [30] and cultural differences and approaches to equity in health, there is a dire need for more voices represented and contributing to these discussions in shaping global policy discourse, measurement, and ultimately, action.

An analysis of authors’ affiliations at the time of publication may demonstrate a further need for discussions around the WHO’s concept of equity to be interrogated and analyzed by those based in the organization, particularly at other regions of the WHO outside of the PAHO, who may yield a novel perspectives by nature of being privy to internal discussions.

In addition, from the articles included in this scoping review, only one had a research methods section [12], despite referring to itself as an essay. This is noteworthy, as it highlights the dire need for empirical research investigating how the WHO has conceptualized equity.

Given that only seven articles in this review were published from 2009 and onwards, when renewed attention to equity was afforded following the CSDH (which convened from 2005 to 2008), it is important to analyze the WHO’s more recent conceptualizations of equity. Similarly, to determine if this differs from the prior approaches and normative positions held by the WHO, particularly following the Alma Ata Declaration in 1978, where “health for all” was coined.

Evidently, the WHO’s discussions of equity (extending beyond Whitehead’s formative definition) are *highly* ambiguous, inadequate, and contradictory, which has led some scholars to question the normative position held by the WHO. While the WHO has expressed alignment with Sen’s Capabilities Approach, this does not seem to be the case in assessing their statements and actions, as their approaches also align with other approaches and theories of justice (e.g. Rawls’ [23]). Further research is needed to empirically investigate the normative position of the WHO. In addition, the WHO should consider striving for reconsideration of the normative and theoretical approaches to equality employed and ensuring consistency.

## Funding

### Sources of funding for the included sources of evidence

From the collated sources included in this review, six declared funding: Asada [20] who reported “This project was supported by grant number 1 R03 HS 13116 from the Agency for Healthcare Research and Quality, and the Canadian Institute of Health Research Training Program for Ethics and Health Policy and Research”; Borde and Hernández [12] who reported “this work was supported by Departamento Administrativo de Ciencia, Tecnología e Innovación: [grant number Colciencias 727]”; Ridde, Guichard [31] who reported “Valéry Ridde holds a Fellowship in Global Health Research Initiative from the Canadian Institutes of Health Research (FGH-81585)”; Smith [34] who reported “This work was supported by Fondation Brocher, a Canadian Institutes of Health Research Frederick Banting and Charles Best Canada Graduate Scholarship, and the Lupina Foundation’s Comparative Program on Health and Society at the Munk School of Global Affairs, University of Toronto”; Smith and Normand [35] who reported “This research was funded by the Irish Research Council for the Humanities and Social Sciences”; and Daniels, Kennedy [24] who reported “Norman Daniels, Bruce P. Kennedy, and Ichiro Kawachi are recipients of Robert Wood Johnson Foundation Investigator Awards in health policy research”.

### Sources of funding for the scoping review

The authors received no specific funding for this work.

## Data Availability

Not applicable/Data sharing is not applicable to this article as no datasets were generated or analysed during the current study.

## Abbreviations

CSDH: Commission on the Social Determinants of Health
PAHO: Pan American Health Organization
PRISMA-ScR: PRISMA Extension for Scoping Reviews
UNICEF: United Nations Children’s Fund
WHO: World Health Organization

## Footnotes

1 32. Ridde V, Guichard A, Houeto D. Social inequalities in health from Ottawa to Vancouver: action for fair equality of opportunity. Promot Educ. 2007;Suppl 2:12–6, 44–7. point to the usage of the term “l’égalité” (equality) in the French translation of the Charter, as opposed to equity in the English version.

2 Please note: these documents were not included in the scoping review as they did not fit the inclusion criteria.
